# Limbic Perfusion Is Reduced in Patients with Myalgic Encephalomyelitis/Chronic Fatigue Syndrome (ME/CFS)

**DOI:** 10.3390/tomography7040056

**Published:** 2021-11-01

**Authors:** Xia Li, Per Julin, Tie-Qiang Li

**Affiliations:** 1 Institute of Information Engineering, China Jiliang University, 258 Xueyuan Street, Xiasha Higher Education Zone, Hangzhou 310018, China; sdlixia@126.com; 2 Department of Neurobiology, Care Sciences and Society, Karolinska Institutet, S-17177 Stockholm, Sweden; per.julin@ki.se; 3 Department of Clinical Science, Intervention and Technology, Karolinska Institutet, S-17177 Stockholm, Sweden; 4 Department of Medical Radiation and Nuclear Medicine, C2-76, Karolinska University Hospital, Huddinge, S-14186 Stockholm, Sweden

**Keywords:** chronic fatigue syndrome, regional cerebral blood flow, pseudo-continuous arterial spin labeling, sustained attention, magnetic resonance imaging, limbic system

## Abstract

Myalgic encephalomyelitis/chronic fatigue syndrome (ME/CFS) is an illness characterized by a diverse range of debilitating symptoms including autonomic, immunologic, and cognitive dysfunction. Although neurological and cognitive aberrations have been consistently reported, relatively little is known regarding the regional cerebral blood flow (rCBF) in ME/CFS. In this study, we studied a cohort of 31 ME/CSF patients (average age: 42.8 ± 13.5 years) and 48 healthy controls (average age: 42.9 ± 12.0 years) using the pseudo-continuous arterial spin labeling (PCASL) technique on a whole-body clinical 3T MRI scanner. Besides routine clinical MRI, the protocol included a session of over 8 min-long rCBF measurement. The differences in the rCBF between the ME/CSF patients and healthy controls were statistically assessed with voxel-wise and AAL ROI-based two-sample t-tests. Linear regression analysis was also performed on the rCBF data by using the symptom severity score as the main regressor. In comparison with the healthy controls, the patient group showed significant hypoperfusion (uncorrected voxel wise *p* ≤ 0.001, FWE *p* ≤ 0.01) in several brain regions of the limbic system, including the anterior cingulate cortex, putamen, pallidum, and anterior ventral insular area. For the ME/CFS patients, the overall symptom severity score at rest was significantly associated with a reduced rCBF in the anterior cingulate cortex. The results of this study show that brain blood flow abnormalities in the limbic system may contribute to ME/CFS pathogenesis.

## 1. Introduction

Fatigue is a subjective feeling commonly reported in many neuropsychiatric illnesses, including myalgic encephalomyelitis/chronic fatigue syndrome (ME/CFS) [1], depression, traumatic brain injury (TBI) [2,3,4], and Parkinson disease (PD). According to community and primary care studies [5], 2–11% of the reported disabling fatigue is chronic and lasts at least 6 months. Fatigue is also common in many other chronic illnesses including cardiovascular disease, cancer, inflammatory arthritis, and osteoarthritis. Fatigue contributes substantially to decrements in the quality of life and disability in these illnesses. Although a substantial percentage of the population experience non-pathological short-term fatigue with an identifiable cause, such as exercise, acute febrile illness, or deprivation of sleep, this type of fatigue is self-limited and recovers by resolving the underlying cause [6]. In contrast, pathological fatigue is characterized by profound fatigue that often has no specific identifiable cause, worsens with exertion, and is not eased by rest and sleep. Despite the clear impact of fatigue as a disabling symptom, our understanding of fatigue pathophysiology is still quite limited for most conditions. For example, it was estimated that ME/CFS affects as many as 2.5 million people in the U.S. alone. The pathogenesis of ME/CFS remains poorly understood, and no effective treatment is available for most patients. In recent years, there has been an increased activity in ME/CFS research. ME/CFS has been recognized as a “serious, chronic, complex system disease” and brought much needed legitimacy to patients facing skepticism and questioning from health care providers. Autoimmune activity triggered by infections has been suggested as a cause of ME/CFS [7]. Autoantibodies directed at muscarinic and betaadrenergic receptors have been identified in ME/CFS and might affect the regulation of both central and peripheral small vessels and blood flow [8].

Energy imbalance or poor energy utilization efficiency has been suggested as one of the most important determinants of fatigue, particularly in older individuals. Different mechanisms have been hypothesized to contribute to fatigue, including a decline in mitochondrial function, alterations in brain neurotransmitters, oxidative stress, and inflammation. Multiple studies have focused on the distinction between fatigue and fatigability [9,10,11]. By investigating the correlation between fatigability and neural activity, these studies have provided new insight into the pathophysiology of fatigability in different illness groups [12,13,14,15,16]. The compensatory theory argues that fatigue is a state caused by task load and represents an overload of pathological information from the injured area to higher cognitive mechanisms. The neuropsychiatric hypothesis suggests that fatigue is a trait unrelated to environmental challenges and can be measured behaviorally by applying specific cognitive tasks. Growing evidence from pathologic and neuroimaging studies [9,10,11] of neurologic disorders suggests that impairment within the frontal-striatal network, involved in effort–reward calculation, is critically related to pathological fatigue. For example, MRI studies of patients with multiple sclerosis have identified a number of neural correlates of self-rated fatigue and fatigability, including cortical thickness, lesion burden, or atrophy in the frontal lobes, basal ganglia, and white matter connections between these regions [17]. Chronic inflammation, TBI [18,19], and major depression are known to give rise to fatigue and may serve as models for how underlying impaired physiological processes contribute to fatigue. However, it remains to be clarified if impairment of the same pathway underlies similar fatigue symptoms in different disorders. A better understanding of abnormal brain changes under fatigue will require two key strategies: the development of measurement tools and exploration of novel treatments, which can lead to symptom-modifying improvement.

The lack of objective measurement has been a major hindrance for clinical assessment of pathological fatigue. In recent years, investigators have suggested distinguishing self-rated fatigue from the objective measures of performance decline by defining the latter as fatigability [5,20] and have attempted to develop neuroimaging biomarkers for assessing fatigue and fatigability [18,20,21]. Among other things, studies based on blood oxygen level-dependent (BOLD) functional magnetic resonance imaging (fMRI), arterial spin labeling (ASL), and magnetic resonance spectroscopy (MRS) have been reported [22,23,24,25,26]. In particular, the pseudo-continuous arterial spin labeling (PCASL) technique can provide quantitative measurement of the regional cerebral blood flow (rCBF) with whole-brain coverage and a relatively high signal-to-noise ratio (SNR). Furthermore, it is non-invasive, and repetitive experiments can be carried out. It has been shown that rCBF measurements based on the PCASL technique may be highly sensitive to slow neural activity changes [27,28,29] and can be a useful biomarker for brain function (Xie et al., 2016). ASL was used to investigate the neural correlates of cognitive fatigue effects in a group of healthy controls (HC) during a 20 min psychomotor vigilance task (PVT) [21]. It was reported that the persistent cognitive fatigue effect was significantly correlated with rCBF decline in the right lateralized fronto-parietal attention network, in addition to the basal ganglia and sensorimotor cortices. Furthermore, the baseline rCBF in the thalamus and right middle frontal gyrus prior to the PVT task was found to be predictive of subjects’ subsequent performance decline. Based on these findings, it has been suggested that the rCBF at rest in the attention network might be a useful indicator of performance potential and a marker of the level of fatigue in the neural attention system. However, a more recent ASL study [22] reported that the global brain perfusion of ME/CFS and HC subjects was similar at rest, despite of the fact that rCBF changes of several brain regions associated with memory, goal-oriented attention, and visual function were differentially related to fatigue in ME/CFS patients and HC. It remains to be clarified if a significant difference exists in the baseline rCBF between ME/CFS and HC subjects. In this study, we leveraged a PCASL MRI technique which can provide whole-brain rCBF measurements to investigate if any rCBF difference exists between a cohort of ME/CFS patients and HC subjects. Furthermore, we also explored if the fatigue severity is associated with the rCBF in the brain.

## 2. Materials and Methods

### 2.1. Subjects

A cohort of 31 ME/CSF patients (average age: 42.8 ± 12.4 years, range 20–62, male/female = 4/27) were recruited for this study at the Neurological Rehabilitation Clinic-Stora Sköndal. All patients fulfilled the Canadian Consensus Criteria, the International Consensus Criteria, and the IOM criteria for ME/CFS [30,31,32]. Other medical or psychiatric diseases were excluded by a thorough medical and psychiatric/psychological evaluation administrated by an experienced neurologist with expertise in ME/CFS rehab, including extensive laboratory tests, neuropsychiatric assessment, and routine neuroradiological examinations. ME/CFS symptom severity was rated by each participant using a standardized rating score which is a rating scale of the severity of symptoms from the International Consensus Criteria (ICC) [31] on a 5-degree scale from 0 to 4 (none, light, moderate, severe, very severe) [33].

The participants were also requested to perform self-rated general physical function and fatigue measurements weekly over at least 8 weeks. The self-rating fatigue symptom measures included the fatigue severity scale (FSS), the Hospital Anxiety and Depression Scale (HADS), and the Swedish version of the visual analog scale (VAS) for the EQ-5D standard measure of patients’ health-related quality of life. The basic information of the FSS, HADS, and EQ-VAS scores is briefly summarized in Table 1.

The FSS questionnaire contains 9 statements that attempt to explore the severity of fatigue symptoms. For the FSS assessment [34], subjects were given a questionnaire sheet with 9 statements and asked to read each statement of the questionnaire and choose a number from 1 to 7 that best described their degree of agreement with each statement: 1 indicates strongly disagree and 7 indicates strongly agree. A mean FSS score over 4 indicates fatigue [33,34].

The HADS is a frequently used scale developed to assess psychological distress in non-psychiatric patients [35]. It consists of two subscales for anxiety and depression. The HADS aims to measure symptoms of anxiety and depression and consists of 14 items: 7 items for the anxiety subscale (HADS Anxiety) and 7 for the depression subscale (HADS Depression). HADS Anxiety focuses mainly on symptoms of generalized anxiety disorder, and HADS Depression is focused on anhedonia, the main symptom of depression [36]. Each item is scored on a response scale with four alternatives ranging between 0 and 3.

In addition to the FSS and HADS, the EQ-VAS [37] was used to measure current self-rated state of health. The Swedish version of the EQ-VAS includes 5 items. The EQ VAS is a vertical visual analog scale in which the individual indicates a number between 0 and 100 that best represents their state of health. On the scale, 100 represents the best possible health and 0 represents the worst possible health.

**Table 1:** Summary of the self-rated scores.

Name	Score Range	Items	References
FSS	1–7	9	[38]
HADS	0–4	14	[35,36]
EQ-VAS	0–100	5	[37]

Medications, tobacco, coffee and tea consumption, food and fluid intake, and number of hours of sleep were also recorded. The participants were also asked if they had experienced events that could have altered their responsiveness leading up to the investigation.

A cohort of 48 HC adult subjects (average age: 46.9 ± 12.0 years, range 20–62) were also recruited for this study. All participants were right-handed and native Swedish speakers. They all reported being free of a history of neurological, psychiatric, and cardiovascular diseases. None of the participants reported any use of psychotropic drugs.

### 2.2. Ethical Approvals and Patient Consent

The ethical application (2016/4:/7) for the study was approved by the regional ethical board in Stockholm, and the study was conducted in line with the Declaration of Helsinki. All participants signed informed consent before enrollment into the study protocol. The HC subjects were financially compensated for their participation.

### 2.3. MRI Protocol

All participants including ME/CFS and HC subjects completed at least one session of the approved MRI protocol on a whole-body clinical 3T Magnetom Prisma MRI system (Siemens Medical Solutions, Erlangen, Germany) equipped with a 64-channel phased array head coil. The MRI acquisition protocol included routine clinical anatomical scans, such as T1W MPRAGE, SWI, T2W GRE, T2W, and FLAIR. There was at least one session of PCASL measurement. The PCASL data acquisitions were based on a 2D single-shot GRE echo planar imaging (EPI) technique with a GRAPPA accelerated parallel method (IPAD = 2). The main acquisition parameters included the following: labeling duration = 1600 ms, TE/TR = 16/3500 ms, FOV = 230 × 230, matrix size = 64 × 64, up to 28 axial slices of 6 mm thickness, an inter-slice gap of 0.6 mm, a sampling bandwidth of 2790 Hz/pixel, and an RF excitation flip angle of 90°. Details regarding the design of the PCASL pulse sequence and optimization of the spin labeling pulses have been described previously [39]. To attain a sufficient SNR for the rCBF data, 70 dynamic pairs of tagging and control scans were carried out, and the entire PCASL session lasted 8.5 min, including 6 dummy scans to attain the signal steady state.

The participants’ heads were carefully fixed in the head coil with foam paddings to reduce involuntary head motions. Prior to data acquisition, the participants were instructed to stay still and focus their sight on a white cross projected on a black screen in front of their eyes and not to think about anything special. These instructions were intended to keep the participants awake and minimize visual stimuli. The participants were in the MRI scanner for approximately 45 min.

### 2.4. Image Data Analysis

The post-processing of the pCASL MRI data was performed offline using shell scripts calling C-programs from the Analysis of Functional NeuroImages (AFNI) software package (http://afni.nimh.nih.gov/afni/ (accessed on 19 August 2021)). The main steps included: (1) motion correction by 3D rigid-body image registration; (2) creation of a brain mask and elimination of extracranial signals based on the temporal average image volume of the time series data; (3) voxel-wise rCBF computation according to a previously established formula [40]; (4) brain normalization to align individual rCBF data to the Montreal Neurological Institute (MNI) brain template by using 12-parameter affine transformation and mutual information as a cost function. During the brain normalization, the rCBF results were also resampled to a voxel size of 2 mm.

To detect if there is any significant difference in the cerebral perfusion between the ME/CFS and HC subjects, we performed a voxel-wise two-sample *t*-test with the spatial-normalized rCBF data using the AFNI program 3dttest++. To investigate if any association exists between the rCBF data and fatigue status of the subjects, we also performed voxel-wise linear regression analyses of the rCBF image data versus the subjects’ FSS and EQ-VAS scores, while age and gender were treated as covariate variables by using the AFNI program 3dRegAna. Statistical significance was assessed by using a two-step approach. Firstly, we imposed a voxel-wise threshold, *p* < 0.001 (uncorrected corresponding *t*-score ≥ 3.2), to form the initial cluster candidates. Secondly, we performed permutation simulations without assuming a particular form of probability distribution for the voxel values in the statistic images to estimate the family-wise error rate (FWER) of the brain regions of interest (ROIs) out of the initially detected clusters. Using the detected ROIs with statistical significance at FWER < 0.05 as masks, we evaluated the mean values of the rCBF for the ROIs and prepared scatter plots for each ROI. Besides the data-driven voxel-wise analysis, for validation we also performed model-driven analysis by leveraging the automated anatomical labeling atlas 3 (AAL3). For each participant, we calculated the average rCBF value for each AAL ROI and then performed a two-sample *t*-test for the obtained average rCBF results.

## 3. Results

The self-rated FSS for the cohort of ME/CFS subjects was 6.6 ± 1.2, which is higher than the ICC for ME/CFS diagnosis (≥4.0). The average scores for fatigue and symptom deterioration with exertion from the HADS were 3.1 ± 0.7 and 3.0 ± 0.7, respectively. The average EQ-VAS was 38.6 ± 24.8. As expected, all the self-rated scores confirmed that, in addition to possessing persistent fatigue, ME/CFS subjects’ fatigue status worsens with exertion.

The clinical evaluation of the structural MRI scans by professional neuroradiologists had no remarkable pathological finding to exclude any of them from the study.

The measured rCBF results from the PCASL MRI protocol for the HC subjects are, overall, in good agreement with results from previous studies [41,42]. Histogram analysis of the inter-subject averaged rCBF data for the HC group indicated the histogram can be fitted reasonably well with two overlapping Gaussian functions, with two peak values at 17 ± 12 and 54 ± 25 mL/100g/min corresponding to the average rCBF for gray and white matter, respectively. The relative amplitude of the two peaks was about 1:2.

**Figure 1:**
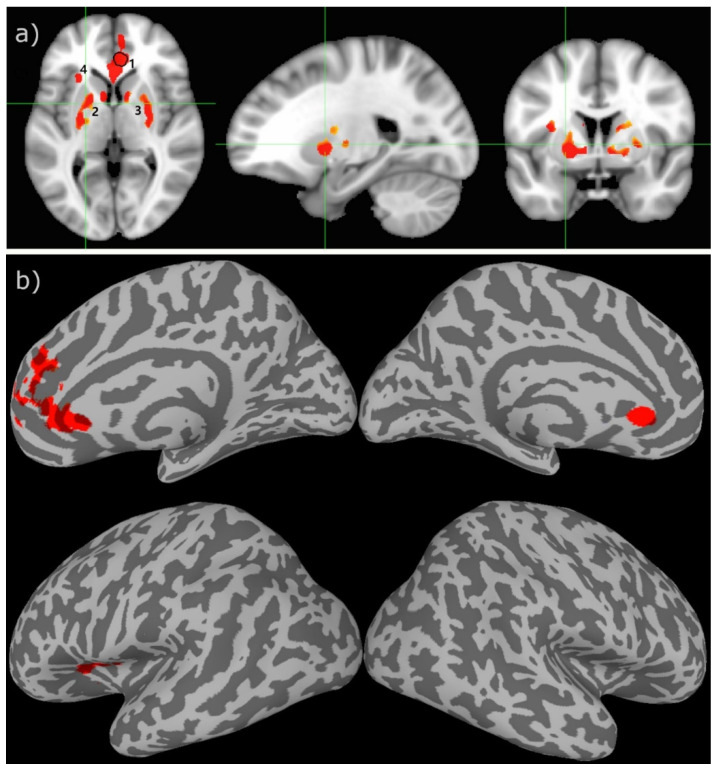
The two-sample *t*-test results between the healthy controls versus the CFS patients overlaid on the MNI152 template in the cross-sectional displays (**a**) and inflated cortical surface (**b**). The green lines indicate the location of cross-sections. The numbers indicate the different ROIs ranked according to their sizes in descending order. The encircled area in ROI 1 indicates the brain region where the subjects’ rCBF values have a significant association with their VAS-f scores. The colored clusters indicate ROIs with t-scores ≥ 3.2 (uncorrected voxel wise *p* ≤ 0.001) and cluster size ≥ 200 voxels (survived FWE correction *p* ≤ 0.01).

**Figure 2:**
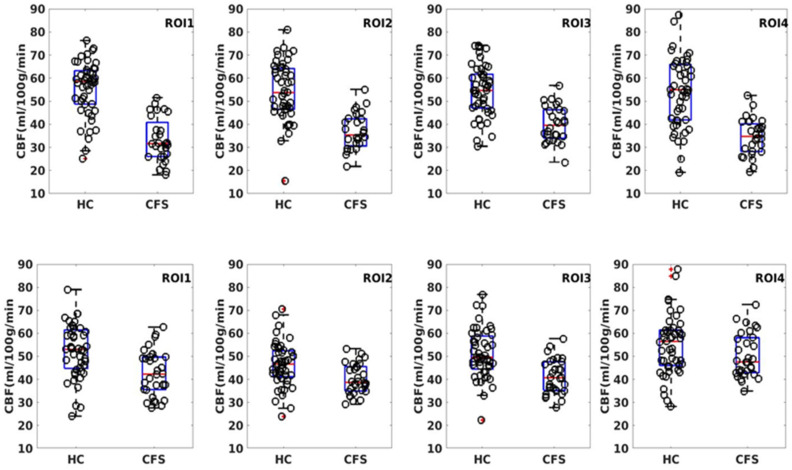
Scattered boxplot of the ROI average for the clusters depicted in Figure 1. The scattered circles indicate the results for individual subjects. The top row depicts the result from the voxel-wise two-sample t-test, whereas the bottom row is the result from the model-driven AAL ROI-based analysis.

The two-sample *t*-test result of the rCBF measurements for the HC and ME/CFS subjects demonstrated that there is statistically significant hypoperfusion (FWER, *p* < 0.01) for the ME/CFS subjects in the limbic system. As shown in Figure 1 and Table 2, the main involved regions are in the limbic system, including the anterior cingulate cortex (ACC), left anterior ventral insular area, pallidum, and caudate nucleus. As depicted in the scattered boxplot (Figure 2), the ROI averaged rCBF for the HC and ME/CFS subjects in these detected regions was 57.9 ± 15.2 and 38.1 ± 8.4 mL/100 g/min, respectively. For the ME/CFS subjects, cerebral perfusion in these brain regions was reduced by about 34% in comparison with the HC group.

**Figure 3:**
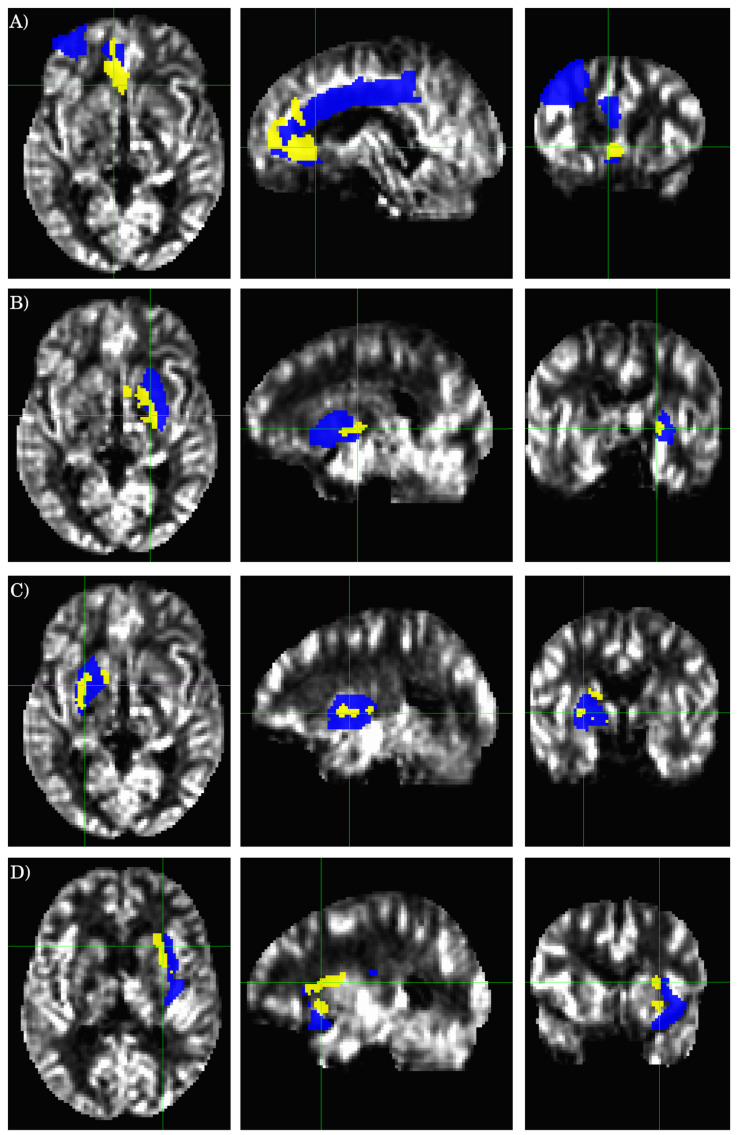
Cross-sectional display of the relevant AAL ROIs and statistically significant clusters as detected by the voxel-wise two-sample *t*-test. The background image is the average rCBF result from the healthy controls. The yellow-colored regions show the clusters detected by the voxel-wise two-sample *t*-test, and the blue-colored regions depict the overlapping AAL ROIs. The crossing green lines indicate the locations of the cross-sections. (**A**–**D**) correspond to the ROIs 1-4 defined in Table 2, respectively.

The results for the AAL ROI-based analysis are summarized in Table 3, and Figure 2 and Figure 3. There is an overall good agreement between the ROI-based analysis and the data-driven voxel-wise analysis. However, the clusters detected by the voxel-wise analysis do not coincide precisely with the atlas-based ROI definition. A cluster can partially overlap with multiple AAL ROIs. AS illustrated in Figure 3A, the largest cluster detected by the voxel-wise analysis (ROI 1 in Table 2), partially overlaps with three different AAL ROIs (AAL ROIs 7, 31, and 33, see Table 3), including the left middle frontal gyrus (AAL ROI 7), the left anterior cingulate (AAL ROI 31), and the left middle cingulate (AAL ROI 33). Similarly, ROI 2 (see Table 2 and Figure 3B) detected by the voxel-wise analysis partially overlaps with the left putamen (AAL ROI 73) and pallidum (AAL ROI 75). ROI 3 (see Table 2 and Figure 3C) detected by the voxel-wise analysis partially overlaps with the right putamen (AAL ROI 74) and pallidum (AAL ROI 76).

There are also notable differences in the results obtained from the voxel-wise and ROI-based analyses. ROI 4 (see Table 2 and Figure 3D) detected by the voxel-wise analysis partially overlaps with the left insula cortex (AAL ROI 29); however, the ROI averaged rCBF values between HC and ME/CFS patients are not significantly different for the entire AAL ROI 29, as assessed by the ROI-based analysis (see Table 3). On the other hand, the voxel-based analysis did not detect any significant cluster in the regions defined by AAL ROIs 43 (L calcarine) and 47 (L lingual gyrus), as expected from the ROI-based analysis results (Table 3).

**Figure 4:**
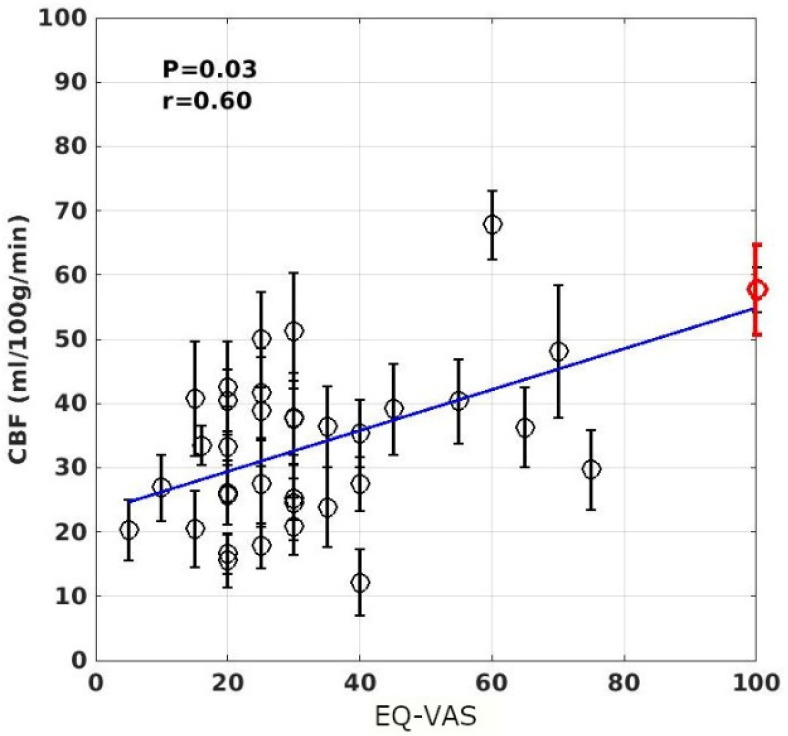
Scatter plot of the ROI averaged rCBF values as a function of the individuals’ EQ-VAS scores. The cluster was detected by voxel-wise linear regression of the rCBF results versus the EQ-VAS scores and using a significance criterion of *p* < 0.05. The error bars indicate the standard errors of the ROI averaged rCBF values for the subjects. The line depicts the linear regression result of the ROI averaged rCBF values versus the individuals’ EQ-VAS scores. The red-colored circled data point at EQ-VAS = 100 indicates the average rCBF result for the HC subjects.

The voxel-wise linear regression of the rCBF data as a function of the individuals’ self-rated FSS and EQ-VAS scores revealed that the rCBF data in a cluster of 87 voxels localized in the ACC (within ROI 1 shown in Figure 1, the coordinate in the MNI152 template space is at (30,31,–6)) are significantly associated with the subjects’ EQ-VAS scores (FWER, *p* < 0.05). The scatter plot of the ROI averaged rCBF data versus the individuals’ EQ-VAS scores is depicted in Figure 4.

## 4. Discussion

The most important findings of this study are the following: (1) There are statistically significant differences in the cerebral rCBF between the ME/CFS patients and adult healthy controls. The involved brain regions are localized in the limbic system, including the ACC, prefrontal cortex, left anterior ventral insular area, pallidum, and caudate nucleus. (2) The ROI averaged rCBF in the ACC is significantly correlated with the ME/CFS patients’ EQ-VAS scores, as detected by voxel-wise linear regression analysis.

In this study, we aimed at investigating the possible abnormality in cerebral perfusion in ME/CFS patients and its association with self-rated fatigue symptoms. The hypoperfusion detected by the two-sample t-test of the rCBF measurements indicates that the ME/CFS patients have an altered cerebral perfusion in several brain regions of the limbic system. Previous studies have also associated neurological abnormalities in these regions with fatigue [21,43,44,45,46]. With the extensive neuroimaging studies of different modalities, a neuroanatomical model of fatigue has gradually begun to emerge. The importance of the non-motor functions of the basal ganglia for explaining mental fatigue was initially proposed about two decades ago [47]. However, later studies realized that the structures in the basal ganglia and thalamus are insufficient to construct a neuroanatomical model to account for fatigue. A network of brain regions mainly from the limbic system including the prefrontal cortex, ACC, insular cortex, amygdala, and nucleus accumbent has been suggested to be associated with fatigue [21,43,45,48]. The superior medial region of the frontal lobe and ACC has been hypothesized to be linked to energizing in attention-demanding tasks [18,19], and dysfunction in these areas might result in slow performance in attention tasks, particularly in procedures that demand the ability to sustain preparation and readiness to respond, such as the psychomotor vigilance task (PVT). The hypoperfusion brain regions associated with ME/CFS patients as detected in the current study coincide precisely with the network which has been suggested to be related to energizing and fatigue. It is, therefore, reasonable to believe that the hypoperfusion associated with ME/CFS subjects might be associated with their cognitive and behavioral abnormalities [10].

As shown in Table 2 and Figure 1 and Figure 3, the brain regions with hypoperfusion are localized in the limbic system, and there is a tendency that the further reduced rCBF is associated with more severe fatigue symptoms, although this association is not significant. The small range of the FSS score might have limited its sensitivity to measure the level of fatigue. In contrast, the range for EQ-VAS is much larger, and it is more likely to provide a finer graded measure for fatigue severity. As discussed above, the EQ-VAS was significantly associated with rCBF for a cluster localized in the ACC (see Figure 1 and Figure 3). In a previous study of mTBI patients with chronic fatigue [18,19], it was also reported that there was a significant association between the self-rated fatigue of post-PVT performance and the rCBF reduction in the ACC. It is well known that the ACC plays an important role in performance monitoring and cognitive control [49]. Hypoperfusion in the ACC can, therefore, lead to impaired monitoring function and cognitive control of the brain. It has been hypothesized that an inability to predict the amount of energy demands for the required performance may underly mental fatigue [43,50]. This implies that the ME/CFS subjects may have an impaired ability to evaluate or adapt to the energy demands to sustain the performance of demanding tasks, which can easily lead to self-rated fatigue.

Cerebral perfusion is an important physiological parameter for brain tissue function, and it is regionally regulated according to regional neuronal activity in the brain. Therefore, detection of microvascular pathological changes may potentially benefit from quantitative assessment of rCBF. With the advent of PCASL techniques, whole-brain perfusion measurement in clinical settings has become practically feasible. PCASL is particularly attractive for clinical applications where repetitive, longitudinal, and quantitative rCBF measurements are desirable. There are several advantages in using the PCASL MRI method for perfusion quantification compared to other techniques such as PET. The technique is non-invasive and does not generate any radiation dose in the studied subjects. The PCASL technique also has relatively high temporal and spatial resolutions. The current major limitation with the PCASL technique is its limited SNR efficiency, even though it is the most efficient among other arterial spin labeling (ASL) techniques. This may change in the future with further development of the technique, such as 3D acquisition. SNR improvement would render rCBF measurements more reproducible and sensitive to pathological abnormalities. The inter- and intra-subject variabilities of rCBF measurement are important issues for its clinical applications. Several groups [51,52,53,54,55] have previously studied the reliability of various ASL techniques. However, most of the previous studies on the reducibility of ASL measurements were based on ROI analysis. Voxel-based analysis of rCBF measurements is very clinically relevant, as demonstrated in the current study, to avoid predefined models and ROIs. All voxels within a predefined AAL ROI are not necessarily included into a cluster with pathophysiological change. Therefore, methods based on a predefined ROI atlas can potentially affect the accuracy and sensitivity of ROI-based analysis. On the other hand, data-driven voxel-wise approaches are dependent on the underlying statistical hypothesis and the distribution of the measurement errors in the data. The use of both model- and data-driven methods may provide mutual validation of the findings.

## 5. Conclusions

Neurological and cognitive aberrations have been well documented for ME/CFS patients; however, relatively little is known about their abnormalities in cerebral perfusion, which is a fundamentally important physiological biomarker for normal brain function. The results from the current study based on a cohort of ME/CFS patients and adult HC subjects demonstrate that there was a statistically significant difference in the cerebral rCBF between the ME/CFS and HC subjects. The involved hypoperfusion brain regions were mainly localized in the limbic system, including the ACC, prefrontal cortex, left anterior ventral insular area, pallidum, and caudate nucleus. Furthermore, the limbic hypoperfusion tended to be associated with their self-rated EQ-VAS scores, although a significant correlation was only detected in a smaller cluster within the ACC. Quantitative measurement of the rCBF with the PCASL technique is useful for studying neurological abnormalities in ME/CFS patients. The experimental evidence from this study supports the notion that brain network abnormalities in the limbic system may contribute to ME/CFS pathogenesis.

## Figures and Tables

**Table 2:** Summary of the ROIs with a significant rCBF deficit in CFS patients in comparison with the healthy controls.

ROI	Voxels	x	y	z	*P*_FWE_	Annotation
1	703	−5.0	−35.1	+6.6	0.01	Anterior cingulate cortex
2	332	+16.8	−1.9	+3.2	0.01	L caudate nucleus/Pallidum
3	259	−20.5	−1.5	+4.8	0.01	R caudate nucleus/Pallidum
4	249	+29.2	−15.7	+7.9	0.01	L anterior ventral insular area

**Table 3:** Summary of the model-driven AAL ROI-based analysis.

AAL ROI N0	HC Average ± Std	CFS Average ± Std	*t*-Score	*p*
7	42.5 ± 13.1	35.1 ± 10.5	2.7	<0.01
31	58.7 ± 14.3	44.0 ± 10.6	4.9	<0.01
33	57.9 ± 14.5	48.7 ± 12.5	2.9	<0.01
43	56.1 ± 13.7	46.7 ± 9.4	3.4	<0.01
47	54.8 ± 13.1	47.4 ± 9.5	2.8	<0.01
73	48.1 ± 10.6	42.0 ± 7.3	2.7	<0.01
74	45.9 ± 10.8	39.3 ± 7.1	3.0	<0.01
75	45.1 ± 9.8	38.2 ± 7.1	3.4	<0.01
76	56.4 ± 11.2	43.1 ± 9.3	5.5	<0.01
30	55.8 ± 14.4	50.3 ± 9.6	1.9	0.07

## Data Availability

The data presented in this study will not be openly available for now due to ethical permission issue.
